# Impact of cemiplimab treatment duration on clinical outcomes in advanced cutaneous squamous cell carcinoma

**DOI:** 10.1007/s00262-024-03728-z

**Published:** 2024-06-08

**Authors:** Domenico Mallardo, Francesca Sparano, Maria Grazia Vitale, Claudia Trojaniello, Mario Fordellone, Eleonora Cioli, Assunta Esposito, Lucia Festino, Mario Mallardo, Vito Vanella, Bianca Arianna Facchini, Rosaria De Filippi, Paolo Meinardi, Margaret Ottaviano, Corrado Caracò, Ester Simeone, Paolo Antonio Ascierto

**Affiliations:** 1 Melanoma, Cancer Immunotherapy and Development Therapeutics Unit, Istituto Nazionale Tumori – IRCCS – Fondazione ‘G. Pascale’, Naples, Italy; 2 Universitiy of Campania “Luigi Vanvitelli”, 81100 Naples, Italy; 3 Department of Clinical Medicine and Surgery, University of Naples Federico II, Naples, Italy; 4 Division of Surgery of Melanoma and Skin Cancer, Istituto Nazionale Tumori ‘Fondazione Pascale’ IRCCS, Naples, Italy

**Keywords:** Cutaneous squamous cell carcinoma, Immune checkpoint inhibitors, Anti-PD1, Duration of response, Immunotherapy, Cemiplimab

## Abstract

Treatment duration with checkpoint inhibitors must be optimized to prevent unjustified toxicity, but evidence for the management of cutaneous squamous cell carcinoma is lacking. A retrospective study was performed to evaluate the survival of patients with cutaneous squamous cell carcinoma (CSCC) who discontinued cemiplimab due to different causes and without progression. Among 95 patients with CSCC who received cemiplimab, 22 (23%) patients discontinued immunotherapy due to causes other than progression, such as comorbidities, toxicity, complete response or lack of compliance (group that discontinued before censoring [DBC]), then 73 patients had standard treatment scheduled (STS). The overall survival was 25.2 months (95% CI: 8.9–29.4) in STS group and 28.3 months (95% CI: 12.7–28.3) in the DBC group; deaths for all causes were 11/22 (50%) in the DBC group and 34/73 (46.6%) in the STS group (*p* = 0.32). 10/22 (45.4%) subjects died due to CSCC in the DBC after discontinuation and 34/73 (46.6%) in the STS group, and the difference between groups was not significant (*p* = 0.230). Duration of treatment was significantly lower in subjects with stable disease versus those with complete or partial response (16.9, 30.6 and 34.9 months, respectively; *p* = 0.004). Among the 22 STS patients, 12 received cemiplimab for less than 12 months (10 [83%] died) and 10 for at least 12 months (1 [10%] died). Our observation, finding no outcome difference between DBC and STS groups, suggests that ICI treatment after one year might expose patients to further treatment related events without efficacy advantages.

**Graphical abstract:**

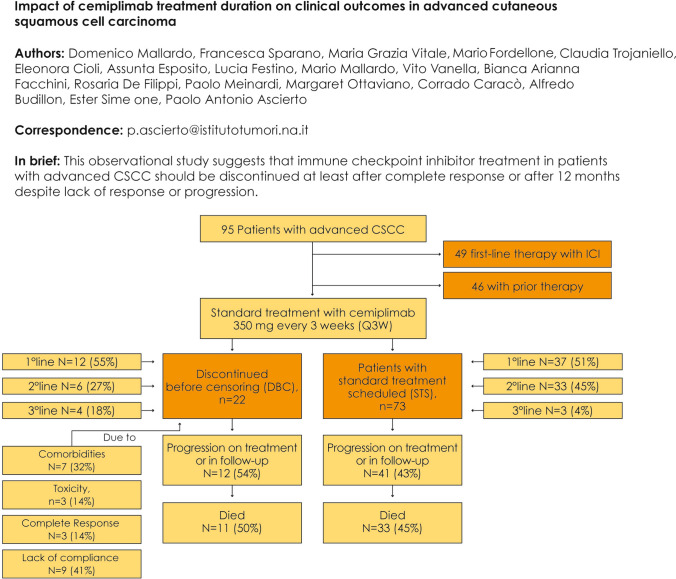

**Supplementary Information:**

The online version contains supplementary material available at 10.1007/s00262-024-03728-z.

## Introduction

The treatment efficacy of malignant tumors has been greatly improved by the introduction of immune checkpoint inhibitors (ICIs), including programmed cell death protein-1 (PD-1)/programmed cell death-ligand 1 (PD-L1) and cytotoxic T-lymphocyte-associated protein 4 (CTLA-4) monoclonal antibodies [1]. Although immunotherapy has provided longer overall survival (OS), ICI use has been associated with immune-related adverse events, as well as high costs, obliging oncologists to explore optimal therapy management in clinical practice and identify the treatment duration that provides the best benefit [2–6].

Some studies on advanced melanoma treated with ICIs have suggested that treatment until progression is not justified [1, 7–10]. Indeed, objective response to nivolumab in patients with advanced melanoma treated for 2 years appears durable after discontinuation [10]. In the KEYNOTE-001 trial evaluating pembrolizumab in advanced malignant melanoma, the 2-year progression-free survival (PFS) after complete response (CR) was not different whether the treatment was stopped or continued [7]. Additionally, data from the KEYNOTE-006 study supported the discontinuation of pembrolizumab in patients with melanoma after 2 years [8]. Notably, rechallenge with a PD-1 blocker following early discontinuation and upon recurrence is effective, as an overall response rate (ORR) of up to 90% has been obtained in malignant melanoma [9]. Studies are currently ongoing to evaluate the impact of stopping ICIs in patients with advanced melanoma upon achieving response: STOP-GAP (NCT02821013) [11] and Safe Stop-T (NTR7502) [4], PET-Stop (NCTN04462406; https://clinicaltrials.gov/study/NCT04462406), and the DANTE study [12]. Based on available evidence, current guidelines for the management of melanoma issued by ASCO recommend stopping immunotherapy after 2 years [13].

Indeed, generalization of these results to any solid tumor is not possible, and evidence for the role of ICI discontinuation before progression of cutaneous squamous cell carcinoma (CSCC) is lacking. To address this issue that may affect clinical practice, a retrospective cohort study was performed to evaluate the survival of patients with CSCC who discontinued cemiplimab without progression due to different causes.

## Patients and methods

### Study design

A retrospective study was carried out at the Istituto Nazionale Tumori—IRCCS—Fondazione “G. Pascale,” Naples, Italy, upon communication to the local Ethics Committee [protocol n. 37/22 oss.]. The study was performed in accordance with the revised version of the Declaration of Helsinki (52nd WMA General Assembly, Edinburgh, Scotland, October 2000).

Consecutive adult patients, aged over 18 years, with advanced CSCC (including metastatic CSCC and locally advanced CSCC not amenable to curative surgery or curative radiation), treated with the anti-PD-1 monoclonal antibody cemiplimab, in either line of treatment, were enrolled between February 2019 and September 2022. All patients provided written informed consent.

### Evaluation of outcomes

RECIST 1.1 criteria were used to assess tumor response as complete response (CR), partial response (PR), stable disease (SD), or progressive disease (PD). The following parameters were collected: duration of treatment, causes of treatment discontinuation, comorbidities, toxicity, response rate at first evaluation, PFS, OS, disease control rate (defined as the sum of CR, PR and SD > 1 year) and ORR (defined as the sum of CR and PR).

### Statistical analysis

Demographic and clinical data were tabulated using descriptive statistics. PFS was calculated from the start of cemiplimab treatment until evidence of PD or death, whichever occurs first; OS was calculated from the start of cemiplimab treatment to death or censoring at the last follow-up. Disease-specific survival (DSS) was calculated as the probability of survival, censoring noncancer causes of death. Survival times were analyzed using the Kaplan–Meier method, and the log-rank test assessed differences among the curves. Using a Cox regression model, hazard ratios (HRs) and their 95% CIs were estimated. Two groups of patients were compared: STS and DBC groups. Cox regression model was used for multivariate and univariate analyses. Duration of treatment was evaluated by stratifying patient for CR, PR and SD. Data are available in 10.5281/zenodo.8402192.

## Results

### Patients’ characteristics

Overall, 95 patients were enrolled, of whom 27 (28%) were females and 68 (72%) were males, with a higher frequency of males as expected for CSCC patients. The median age was 75 years, ranging from 32 to 96 years. The therapy used before cemiplimab was chemotherapy in 32 (34%) of the cases, chemotherapy + radiotherapy in 10 (11%), immunotherapy in 1 (1%), and targeted therapy in 2 (2%). Nine patients had metastases invading the skullcup and the leptomeningeal membrane at baseline, brain metastases were not detected in any subject.

All patients were planned to receive cemiplimab 350 mg every 3 weeks until disease progression or unacceptable toxicity, according to the recommended schedule [14]. At the time of censoring, 22 (23%) patients had discontinued cemiplimab due to causes other than progression (DBC group) and 73 patients were in standard treatment (STS group). In the DBC group, seven (32%) subjects discontinued cemiplimab due to comorbidities, three (14%) due to toxicity, three (14%) due to CR, and nine (41%) due to lack of compliance with therapy. The overall survival (OS) was 25.2 months (95% CI: 8.9–29.4) in the STS group, and 73 (77%) patients were in the STS group.

The patients’ characteristics and comorbidities at baseline are shown in Table 1.

**Table 1:** Demographic and clinical characteristics at baseline, in the entire study population

Patient characteristics	DBC group (n = 22), n (%)	STS group (n = 73), n (%)	Total (n = 95), n (%)
Age, median (range)	74 (32–96)	77 (55–95)	75 (32–96)
*Gender:*			
Female	3 (14)	24 (33)	27 (28)
Male	19 (86)	49 (67)	68 (72)
*ECOG performance status:*			
0–1	15 (68)	49 (67)	64 (67)
≥ 2	7 (32)	24 (33)	31 (33)
*Desease level:*			
Locally advanced	11 (50)	36 (49)	47 (50)
Nodal metastasis	6 (27)	15 (21)	21 (22)
Distant metastasis	5 (23)	22 (30)	27 (28)
CSCC invading skullcap and leptomeningeal membrane	0 (0)	9 (12)	9 (9)
*Line of treatment with anti-PD-1:*			
First-line treatment	12 (55)	37 (49)	49 (52)
Second-line treatment or more	10 (45)	36 (51)	46 (48)
*Type of previous therapy:*			
Chemotherapy	5 (23)	31 (42)	36 (38)
Chemotherapy + radiotherapy	5 (23)	5 (7)	10 (11)
*Response rate at first assessment:*			
Complete response	3 (14)	7 (10)	10 (11)
Partial response	1 (5)	24 (33)	25 (26)
Stable disease	10 (45)	25 (34)	35 (37)
Progressive disease	9 (41)	16 (22)	25 (26)
Overall response rate	4 (18)	31 (42)	35 (37)
Disease control rate	11 (50)	27 (37)	38 (40)
*Comorbidities*			
Myocardial infarction	0 (0)	7 (10)	7 (7)
Angina/coronary artery disease	4 (18)	6 (8)	10 (10)
Arrhythmias	4 (18)	11 (15)	15 (16)
Hypertension	10 (45)	26 (36)	36 (38)
Respiratory system disease	2 (9)	2 (3)	4 (4)
Hepatic disease	2 (9)	0 (0)	2 (2)
Renal system disease	2 (9)	2 (3)	4 (4)
Diabetes	4 (18)	9 (12)	13 (14)
Neuromuscular disease	2 (9)	0 (0)	2 (2)
Leukemia and myeloma	1 (5)	1 (1)	2 (2)
Malignancy other than leukemia and myeloma	2 (9)	5 (7)	7 (7)
Dyslipidemia	3 (14)	2 (3)	5 (5)
Others	6 (27)	16 (22)	22 (23)

*DBC* discontinued before censoring, *STS* standard treatment scheduled

In the overall sample, cemiplimab was administered as the first-line treatment to 49 (52%) patients and second- or further-line treatment to 46 (48%).

In the DBC group, cemiplimab was administered first-line in 12 (55%) of these patients, second-line in six (27%), and third-line in four (18%).

**Fig. 1:**
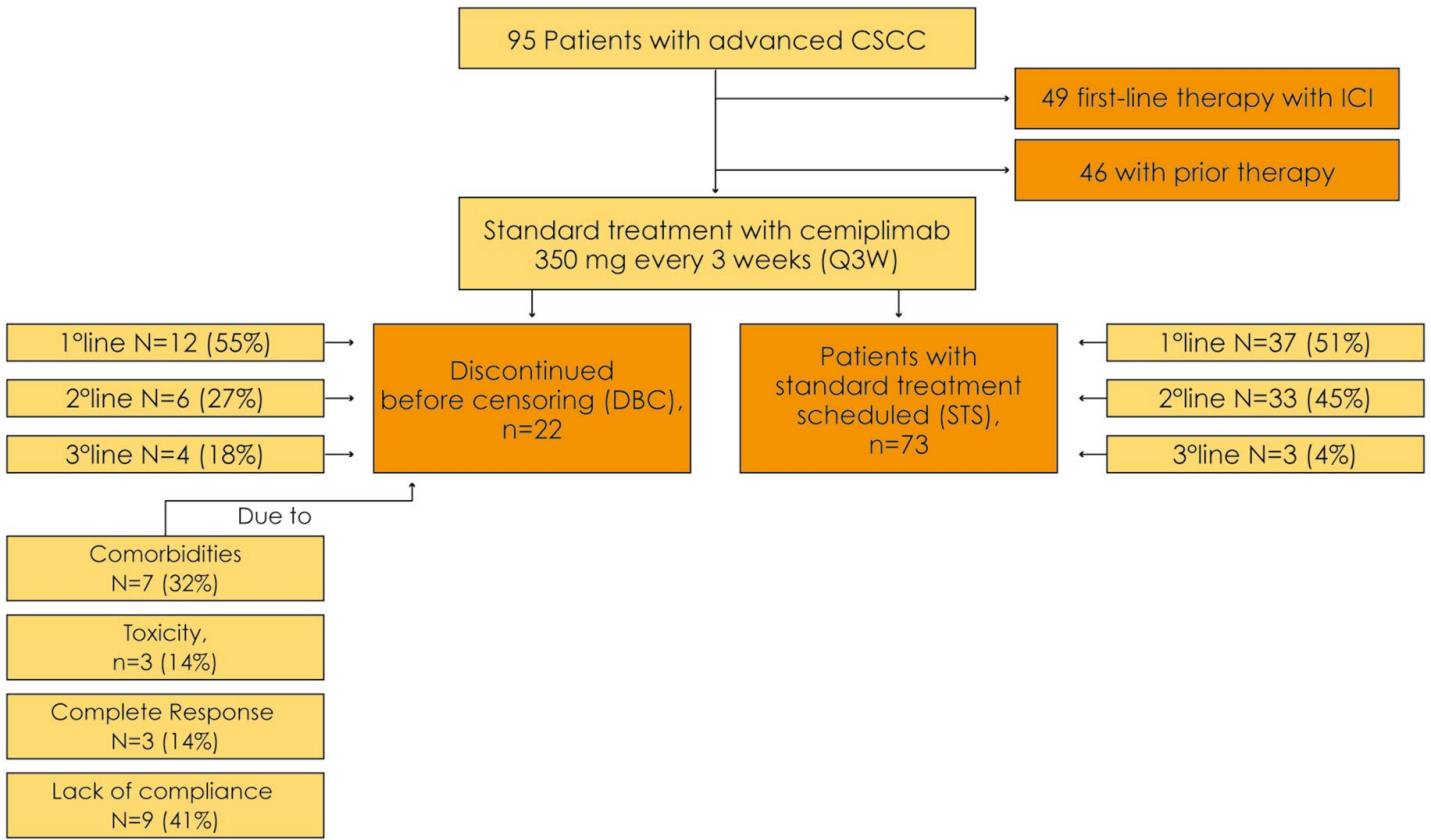
Treatments and disposition of cutaneous squamous cell carcinoma patients included in the study. ICI: immune checkpoint inhibitors; CSCC: cutaneous squamous cell carcinoma

Among the 73 (77%) STS group, 44 (60%) had discontinued cemiplimab due to PD, and 29 (40%) were continuing the treatment. In these patients, cemiplimab was administered in the first line in 37 (51%) of these patients, second line in 33 (45%), and third line in three (4%) (Fig. 1).

**Fig. 2:**
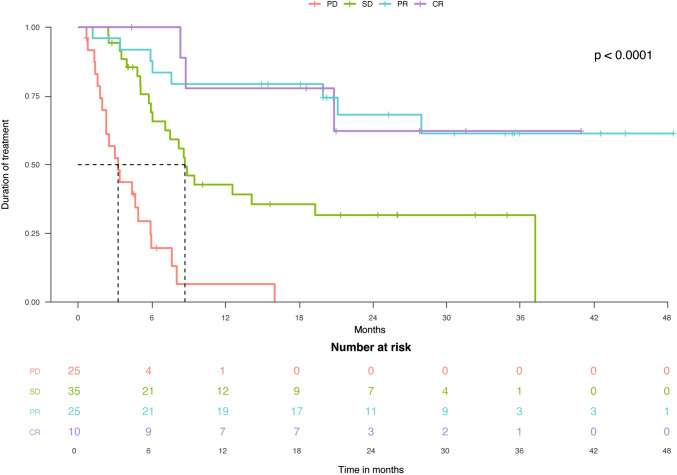
Duration of treatment according to response to treatment. CR: complete response; PR: partial response; SD: stable disease; PD: progression disease

The median duration of treatment with cemiplimab was 27.9 months (95% CI: 9.5–37.2) [mean = 26.9 months (95% CI: 21.8–32.0)] in the overall sample, 8.7 (95% CI: 5.9–37.2) [mean = 16.9 months (95% CI: 11.7–22.1)] in those with SD, and was not reached in patients with CR or PR (mean = 30.6 months (95% CI: 21.1–40.0) in patients with CR, 34.9 months (95% CI: 27.2–42.5) in those with PR). Duration of treatment was significantly lower in subjects with SD versus those with CR or PR (*p* = 0.004) (Fig. 2).

### Treatment outcomes

After 3 months from starting treatment with cemiplimab, among the entire study population, 10 (11%) patients had obtained CR, 25 (26%) PR, 35 (37%) SD, and 25 (26%) PD. The ORR was 35 (37%), and the overall disease control rate was 38 (40%). Disease control rate was 50% in the DBC group and 37% in the STS group.

**Fig. 3:**
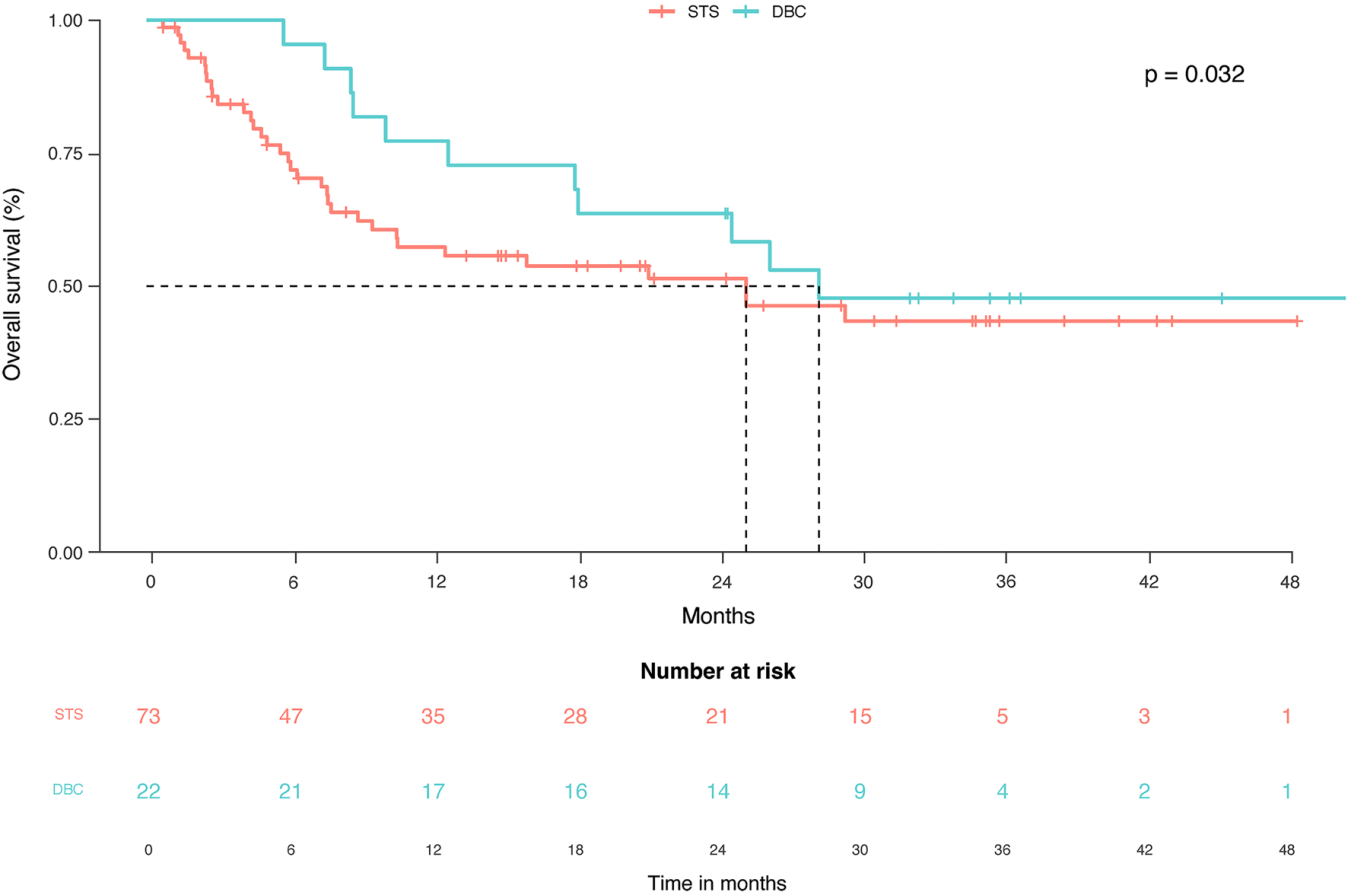
Overall survival in the whole population and in the subgroup that discontinued cemiplimab without progressive disease. DBC: discontinued before censoring; STS: standard treatment scheduled

The median OS at censuring was 25.2 months (95% CI: 8.9–29.4) in the STS group and 28.3 months (95% CI: 12.7–28.3) in the DBC group. In the overall population, 45/95 (47.3%) patients had died of any cause, 11 in the DBC group (HR: 1.37; 95% CI: 0.7–2.5) and 34 in the STS group (HR: 0.72; 95% CI: 0.3–1.3). Therefore, the proportion of subjects who died in the DBC group (11/22, 50%) and in the STS group (34/73, 46.6%) was not significantly different (*p* = 0.32) (Fig. 3). In addition, multivariate and univariate analyses showed no significant difference (Supplementary Table 2). All deaths were due to the CSCC, except one due to COVID-19.

**Fig. 4:**
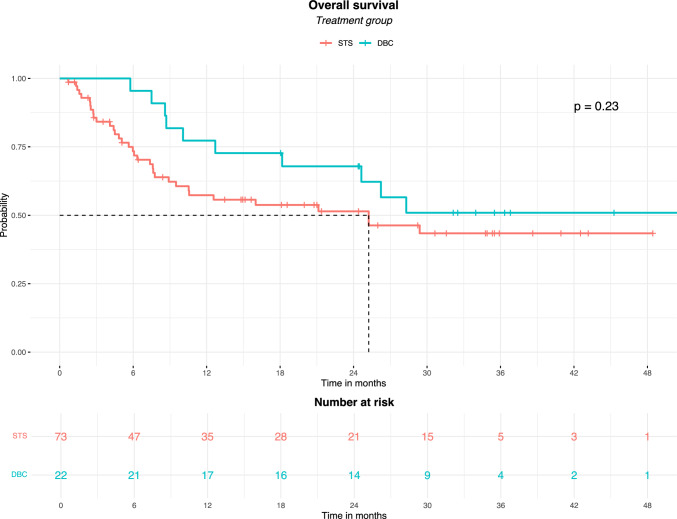
Disease-specific survival in the standard treatment scheduled and discontinued before censoring groups. DBC: discontinued before censoring; STS: standard treatment scheduled

The median DSS was 25.2 months (95% CI: 8.9–29.4) in the STS group, was not reached in the DBC group. Indeed, 10/22 (45.4%) subjects died due to CSCC in the DBC group and 34/73 (46.6%) in the STS group, and the difference between groups was not significant (*p* = 0.230) (Fig. 4).

**Fig. 5:**
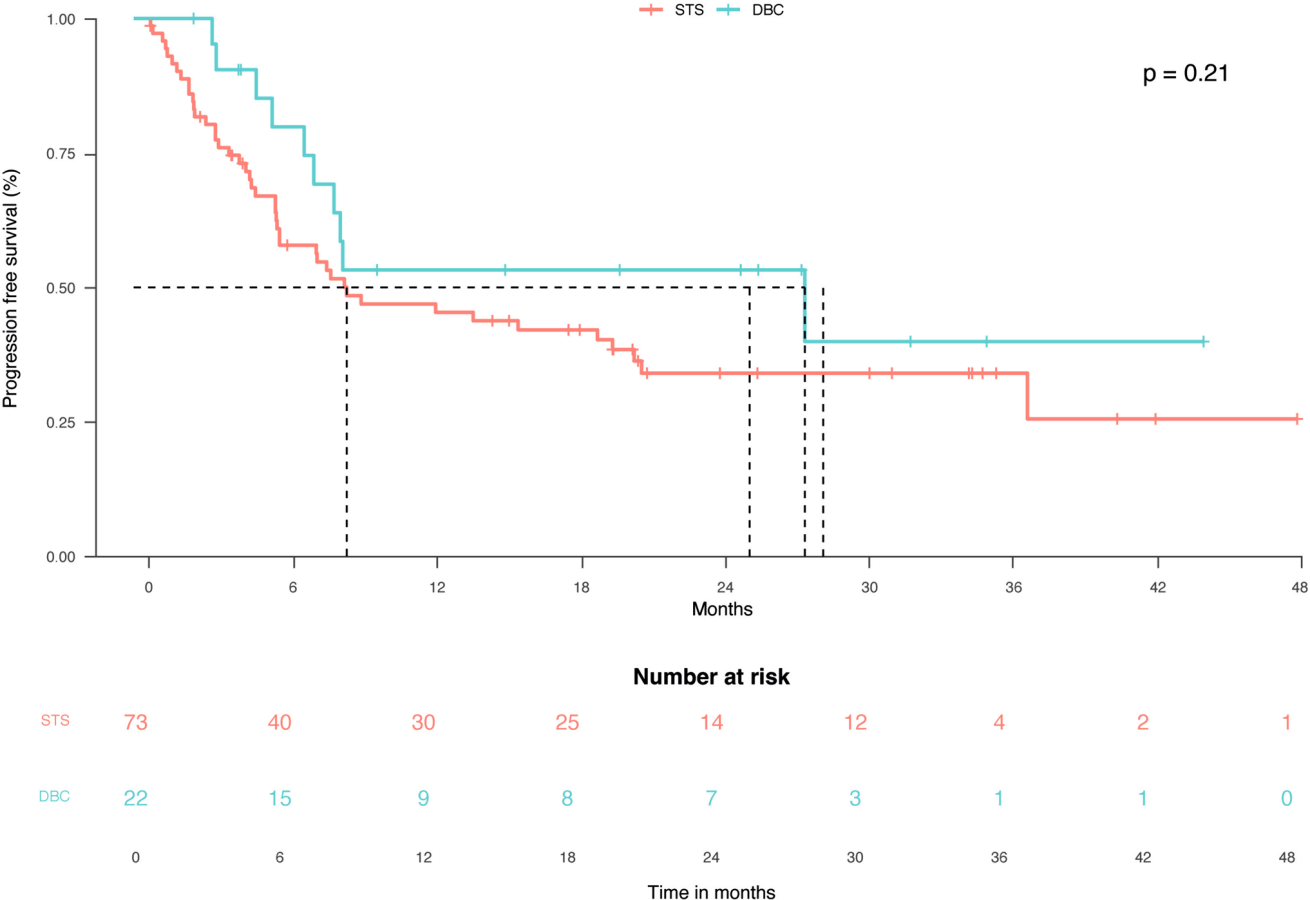
Progression-free survival in the standard treatment scheduled and discontinued before censoring groups. DBC: discontinued before censoring; STS: standard treatment scheduled

**Fig. 6:**
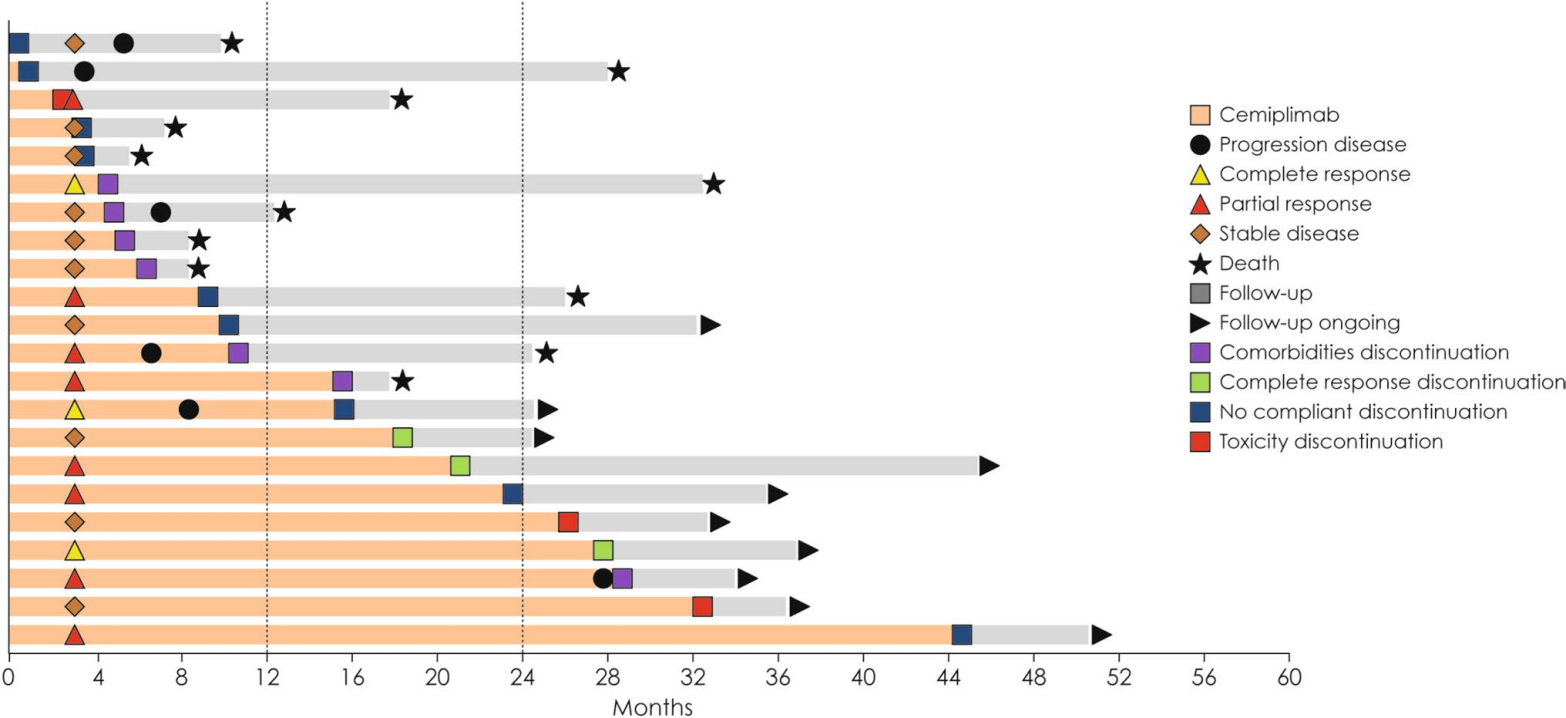
Clinical course of the 22 patients in the discontinued before censoring group

The median PFS at censuring was 8.8 months (95% CI: 7.5–21.1) in the whole population, 27.9 months (95% CI: 7.1–27.9) in the DBC group, and 8.8 months (95% CI: 5.9–20.8) in the STS group. In the whole population, 54/95 (41%) patients experienced a progression, 10/22 subjects (45.4%) had PD at censoring among those in the DBC group (HR: 1.4; 95% CI: 0.8–2.7), and 44/73 (60.3%) in the STS group (HR: 0.6; 95% CI: 0.3–1.2); the difference between the DBC group and the latter group was not significant (*p* = 0.21; Fig. 5). Univariate and multivariate analyses showed no differences (Supplementary Table 2). Figure 6 shows the clinical course of the 22 patients in the DBC group. Among these patients, 12 received cemiplimab for less than 12 months and 10 for at least 12 months. Among the former ones, 10 (83%) died, while among the latter ones, only 1 (10%) died. Five patients received cemiplimab for at least 24 months and were all alive at study censoring.

One patient had obtained CR within 4 months and discontinued the treatment for comorbidity at month 5: he had a durable response, being followed up to month 32. Three patients who similarly discontinued for comorbidities at 4–6 months but with SD died at month 8 in two cases and at month 13 in one case.

Three patients discontinued for toxicity: one at month 4 and died at month 18; two after more than 24 months of treatment, and both had durable SD at month 36.

## Discussion

This retrospective study evaluated the clinical outcomes of patients with advanced CSCC who were treated with cemiplimab and discontinued therapy for causes other than disease progression. Overall, we observed that OS, DSS and PFS were not different in those that discontinued before censored (DBC group) and those with standard treatment scheduled (STS group). As the two groups had similar characteristics, for age, ECOG, and comorbidities, our results suggest that prolonged exposure to treatment may not be beneficial, increasing the risk of adverse events without improving survival [15, 16].

In the DBC group, the proportion of those who died was higher when cemiplimab had been discontinued before 12 months (83% died) than when it was discontinued after 12 months (10% died), and patients who discontinued cemiplimab after 12 months of treatment had durable responses up to month 52. These observations could suggest the need to administer at least 12 months of treatment unless CR is obtained, if confounding factors such as comorbidities, general conditions and age could be controlled by a study in a larger cohort.

Four patients discontinued therapy after achieving CR and all had durable responses, suggesting that continuing immunotherapy after CR is not justified. This result appears to agree with observations from the KEYNOTE-001 trial in patients with melanoma [7].

Some data suggest that even very short courses of 3–6 months of ICIs may be sufficient in advanced melanoma once CR or PR is obtained [17]. Although the limited number of patients in this study does not allow conclusions to be drawn, we observed that among patients who discontinued at 4–6 months, only the one who had achieved CR had a durable response, while all others died within 1 year. Thus, PR does not seem to be a sufficient outcome for very early discontinuation.

The analysis of the duration of treatment stratified by response suggested that those who have a response and are treated for 24 months may have durable responses. A study on a larger sample is necessary to confirm that treatment could be stopped after 24 months in responding subjects.

This study had some limitations including the observational retrospective design and the small sample size; in addition, it did not evaluate patient rechallenge with ICIs after recurrence. This retrospective observational study had not a study design but we performed very strict statistical analyses, according to the methods of previous studies [18].

In conclusion, our observation, finding no outcome difference between DBC and STS groups, suggests that ICI treatment after one year might expose patients to further treatment related events without efficacy advantages. Very limited evidence suggests that therapy may safely be discontinued before 12 months. Further investigation into larger populations is necessary to draw definite evidence.

### Supplementary Information

Below is the link to the electronic supplementary material.

Supplementary file1 (DOCX 25 kb)

## Data Availability

10.5281/zenodo.8402192
