# Supplementary material for: Impact of cemiplimab treatment duration on clinical outcomes in advanced cutaneous squamous cell carcinoma
Source: Cancer Immunol Immunother. 2024 Jun 8;73(8):160. doi: 10.1007/s00262-024-03728-z (PMC11162402; doi:10.1007/s00262-024-03728-z)
Supplement: Supplementary file 1 — Supplementary file1 (DOCX 25 kb) [file 262_2024_3728_MOESM1_ESM.docx]

**Supplementary table 2. Univariate and multivariate analyses for OS and PFS**

| **OS** | | | | | | |
| --- | --- | --- | --- | --- | --- | --- |
|  | **Univariate analysis** | | | **Multivariate analysis** | | |
| **Covariate** | **HR** | **95% CI** | **P** | **HR** | **95% CI** | **P** |
| **Age** | **1,0137** | **0,9867 to 1,0414** | **0,3231** | **1,019** | **0,9906 to 1,0482** | **0,1915** |
| **Gender** | **0,9638** | **0,5125 to 1,8127** | **0,9089** | **0,8838** | **0,4518 to 1,7291** | **0,7184** |
| **First line treatment** | **0,8747** | **0,4809 to 1,5911** | **0,6611** | **0,797** | **0,4271 to 1,4874** | **0,4761** |
| **DBC** | **0,7127** | **0,3603 to 1,4097** | **0,3304** | **0,6357** | **0,3125 to 1,2931** | **0,2111** |
| **PFS** | | | | | | |
|  | **Univariate analysis** | | | **Multivariate analysis** | | |
| **Covariate** | **HR** | **95% CI** | **P** | **HR** | **95% CI** | **P** |
| **Age** | **1,0044** | **0,9809 to 1,0284** | **0,7187** | **1,0085** | **0,9836 to 1,0341** | **0,5081** |
| **Gender** | **1,0548** | **0,5796 to 1,9195** | **0,8613** | **1,0082** | **0,5280 to 1,9253** | **0,9803** |
| **First line treatment** | **0,7342** | **0,4263 to 1,2645** | **0,2653** | **0,7167** | **0,4096 to 1,2541** | **0,2433** |
| **DBC** | **0,6479** | **0,3260 to 1,2879** | **0,2157** | **0,6182** | **0,3066 to 1,2463** | **0,1788** |
